# Pregnancy complicated with PFAPA (periodic fever, aphthous stomatitis, pharyngitis and cervical adenitis) syndrome: a case report

**DOI:** 10.1186/s12884-018-1854-6

**Published:** 2018-06-04

**Authors:** Kuniaki Ota, Joanne Kwak-Kim, Toshifumi Takahashi, Hideki Mizunuma

**Affiliations:** 1 Department of Obstetrics and Gynecology, School of Medicine, Fukushima Medical University, 1-Hikarigaoka, Fukushima, 960-1247 Japan; 2 Fukushima Medical Center for Children and Women, School of Medicine, Fukushima Medical University, 1-Hikarigaoka, Fukushima, 960-1247 Japan; 3 Reproductive Medicine and Immunology, Department of Obstetrics and Gynecology, Chicago Medical School, Rosalind Franklin University of Medicine and Science, 830 West End Court, Suite 400, Vernon Hills, IL 60061 USA

**Keywords:** PFAPA, Pregnancy, Th1/Th2, Adult onset, Periodic fever, Aphthous stomatitis, Pharyngitis, Cervical adenitis

## Abstract

**Background:**

Periodic fever, aphthous stomatitis, pharyngitis and cervical adenitis (PFAPA) syndrome has been considered as a childhood syndrome. The underlying etiology of PFAPA syndrome is unclear however, currently considered as auto-immune inflammatory disease. Recently, a few cases of adult-onset of PFAPA syndrome have been reported. However, there is no report about the successful management of pregnancy complicated with PFAPA syndrome.

**Case presentation:**

The patient was a 31-year-old woman who developed recurrent episodes of high fever associated with cervical adenitis, pharyngitis and vomiting started 9 months after a delivery. She was diagnosed with PFAPA syndrome and cimetidine 800 mg/day was initiated. Since then, these symptoms got better. Cimetidine treatment was discontinued since she became pregnant (6 weeks of pregnancy). Except one febrile episode at 8 weeks gestation, she did not develop a febrile episode during pregnancy.

Peripheral blood Th1/Th2 ratio was decreased from the first trimester to the second trimester of pregnancy. Then again, the ratio was steadily elevated during the third trimester. At 38 weeks, she delivered a live born infant without any complication. Two months after delivery, she developed PFAPA syndrome again and cimetidine treatment was re-initiated. However, febrile episodes were not controlled well, and Th1/Th2 ratio was further elevated compared to pregnancy status. Colchicine 0.5 mg once a day was initiated. Symptoms were diminished and Th1/Th2 ratio was gradually decreased.

**Conclusion:**

There was no case report of pregnancy complicated with PFAPA syndrome, though there were several reports of adult-onset PFAPA cases without pregnancy. The current case may be the first case report of a successful pregnancy complicated with PFAPA. In this case, PFAPA symptoms were ameliorated during pregnancy, but reappeared after delivery. We speculate that PFAPA syndrome, a Th1 type immune disorder, might be improved due to the Th1 to Th2 shifting, which was induced by pregnancy. It is necessary to investigate further about PFAPA syndrome with pregnancy and Th1/Th2 immune responses in the future.

## Background

PFAPA (periodic fever, aphthous stomatitis, pharyngitis and cervical adenitis) syndrome which was first reported in 1987, is the most common autoimmune inflammatory fever disorder in childhood worldwide [1]. It is characterized by predictably periodic high fever lasting for approximately 4 days (ranges 2 to7 days) and associated with at least one of three clinical symptoms, such as pharyngitis, cervical adenitis and aphthous stomatitis [2]. The underlying etiology of the disease is still unknown, and the diagnosis is made with the clinical criteria proposed by Thomas et al. [2]; 1) Periodic fevers with an early age of onset (< 5 years of age), 2) Symptoms in the absence of upper respiratory tract infection with at least 1 of the following clinical signs: a) aphtous stomatitis, b) cervical lymphadenitis, c) pharyngitis, 3) Exclusive of cyclic neutropenia, 4) Completely asymptomatic interval between episodes, 5) Normal growth and development. Furthermore, PFAPA is required to exclude other diseases of recurrent fevers in childhood, such as malignancies, autoimmune and infectious disease. Genetic variants of the innate immune system, such as familial Mediterranean fever (FMF), TNF receptor-associated periodic syndrome (TRAPS), mevalonate kinase deficiency (MKD) and cryopyrin-associated periodic syndromes (CAPS) are also included as the differential diagnosis since PFAPA syndrome is currently supposed the pathogenesis of abnormal host immune response [3]. To date, some researchers reported that adult-onset of PFAPA syndrome, though PFAPA syndrome is basically pediatric disease and usually settles in adolescence [4, 5].

We would like to present a case of successful pregnancy complicated with PFAPA syndrome. We believe this is the first report of pregnancy complicated with PFAPA, since PFAPA primarily affects preschool-age children [6] and is rarely occurred in adults [7].

## Case presentation

The patient was a healthy 31-year-old woman who had an uneventful delivery of a live born infant at term 4 years ago. Nine months after the delivery, she developed recurrent episodes of high fever (39 °C) followed by cervical adenitis, pharyngitis and vomiting. A disease-free interval, which ranges 4 to 8 weeks, was observed between the periodic fever episodes, and a menstrual cycle was not related to the onset of a febrile episode.

Elevated C-reactive protein (peak value of 13.9 mg/dL) was noticed. Other laboratory studies, including immunoglobulin levels, serum complement level, immuno-phenotypic characterization of lymphocytes, HIV, CMV and EBV serology, and antinuclear antibodies, were negative. Genetic tests for genomic DNA from whole blood were conducted to exclude FMF, TRAPS, MKD and CAPS and all were negative. Cyclic neutropenia was excluded by serial neutrophil counts.

Her PFAPA symptoms disappeared spontaneously within 5 days without antibiotic treatment and the elevated CRP level became normal in 10 days from the first day of fever. Finally, PFAPA syndrome was diagnosed according to the Padeh’s criteria, which are not restricted to the age of onset [8]. To prevent future febrile episodes, cimetidine 400 mg twice daily was started, and 60 mg of intravenous prednisolone was given during a febrile attack. During the subsequent febrile episodes, PFAPA symptoms were lessened. After 8 months passed, she became pregnant and quit cimetidine at the 6th week of gestation by herself. She then developed only one febrile episode at 8th week of gestation without any subsequent febrile episode during pregnancy, possibly due to altered maternal immunity with advanced pregnancy.

**Fig. 1:**
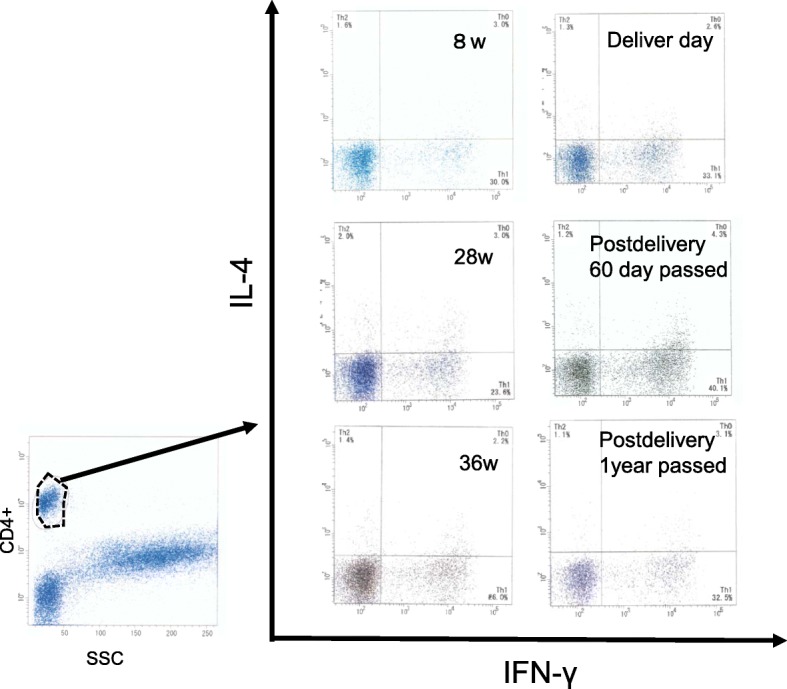
Th1/Th2 cell ratio (IFN-γ/IL-4 T helper cell ratio) was analyzed by flow cytometric analysis. An acquisition gate was established based on CD4 staining and side scatter (SCC) which included peripheral blood mononuclear cells (left). Dot plot analysis of IFN-γ and IL-4 expressing CD4^+^ T cells from the patient. Numbers indicate percent gated cells (right)

**Fig. 2:**
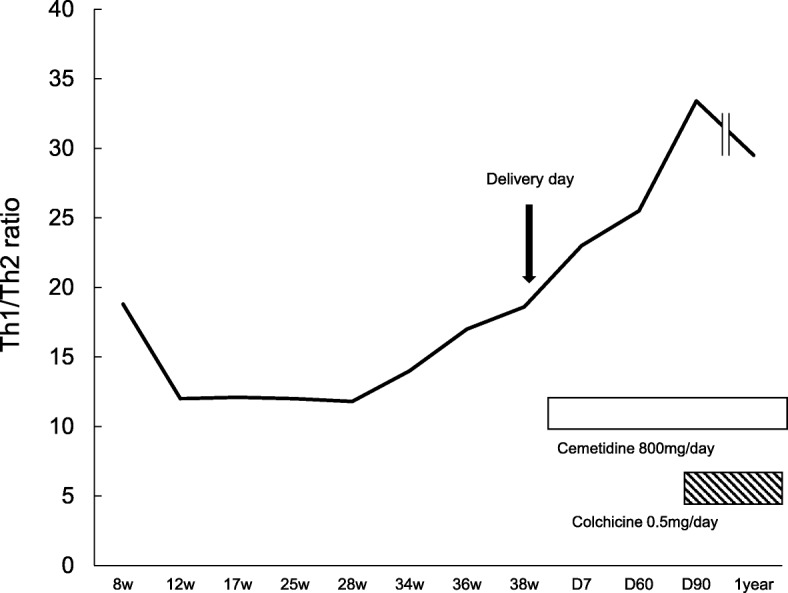
Time course plot of Th1/Th2 cell ratios (IFN-γ/IL-4 T helper cell ratio) from early pregnancy to postpartum 1 year. The white box indicates the period of cimetidine treatment and the slant bow indicates the period of colchicine treatment

T helper (Th)1/Th2 cell ratios were analyzed every 2~ 6 weeks during pregnancy (Fig. 1). IFN-gamma/ IL-4 producing CD3+/CD4+ T helper cell ratios were elevated during the first trimester. During the second trimester, Th1/Th2 ratios were decreased. CRP remained in the normal ranges during entire pregnancy. Whereas Th1/Th2 ratios were increased gradually even without a febrile episode during the third trimester. At 38 weeks, she developed a premature rupture of membrane and delivered a normal healthy male infant, weighing 2873 g. The Apgar scores (1 and 5 min) were 9 and 10 respectively. Two months after delivery, she developed an episode of high fever associated with cervical adenitis, pharyngitis and vomiting as she did prior to pregnancy. Cimetidine was re-initiated. Th1/Th2 ratio was 25.5 (Fig. 1). Febrile episodes became worse in severity and frequency and Th1/Th2 ratio was further elevated to 33.4 one month later. Colchicine 0.5 mg once a day was added. Th1/Th2 ratio started to decrease gradually (Fig. 2). Currently, febrile episodes remain shorter and milder.

## Discussion and conclusions

A case of pregnancy complicated with PFAPA syndrome has not been reported despite some reported adult-onset cases [7]. Although the exact pathogenesis of PFAPA has yet to be elucidated, it is considered as a polygenic autoinflammatory disease in which a microbial trigger might give rise to the activation of innate immune system and recruitment of activated T cells in a susceptible host, leading to Th1 driven immune responses [9]. On the other hand, the predominant Th2-type immunity has been observed during normal pregnancy. Maternal tolerance toward fetal allo-antigens was explained by the predominant Th2-type immunity during pregnancy for protecting the fetus from maternal Th1-immunity [10]. During normal pregnancy, Th1/Th2 ratio increases transiently during implantation period, and then decreases after implantation is over. During the third trimester, Th1/Th2 ratio increases for the preparation of parturition. Persistently increased Th1/Th2 ratios have been associated with multiple implantation failures and repeated pregnancy losses [11, 12]. In this case, Th1/Th2 ratio was elevated during the early first trimester of pregnancy. However, she could succeed in pregnancy and deliver despite the predominant Th1-type immunity which is harmful to pregnancy maintenance. We speculate that PFAPA by Th1 immune disorder might be ameliorated during the second trimester of pregnancy possibly due to the predominant Th2 immunity established during pregnancy. Furthermore, pregnancy complicated with PFAPA can be treated as normal perinatal course since symptoms are relieved by shifted Th2 immunity. It is necessary to pay sufficient attention because febrile episodes are rapidly exacerbated after parturition.

## Abbreviations

CAPS: Cryopyrin-associated periodic syndromes
CD: Cluster of differentiation
CMV: Cytomegalovirus
CRP: C-reactive protein
DNA: Deoxyribonucleic acid
EBV: Ebstein-Barr virus
FMF: Familial Mediterranean fever
HIV: Human immunodeficiency virus
IFN: Interferon
IL: Interleukin
MKD: Mevalonate kinase deficiency
PFAPA: Periodic fever, aphthous stomatitis, pharyngitis, and cervical adenitis
Th1/Th2: T helper 1/T helper 2
TRAPS: TNF receptor-associated periodic syndrome
